# Difference in the Estimate of Longevity

**Published:** 1881-02

**Authors:** 


### DIFFERENCE IN THE ESTIMATE OF LONGEVITY.

A writer in the last number of *The American*, speaking of longevity, observes that there is one point which is too frequently overlooked by writers on historical cases of longevity — the singular difference between our estimation of an old man and that of our forefathers. "Old John of Gaunt, time-honored Lancaster," died at fifty-nine, so did James I; Henry VII only reached fifty-three. The Huguenot Admiral, Coligny, whose "hoary hair, all dabbled o'er with blood'" is mentioned by Macaulay, and whom his earlier biographer, Lord Huntington, represents as a very old man, was slain at fifty-three, when, in these days of Moltkes, Gortschakoffs, Gladstones, Beaconsfields, Dufaures, Broughams, and Lyndhursts, a man is supposed to be rather on the juvenile side. In point of fact, for our ancestors, a man of fifty was old; with us, the limit is nearer eighty. It is legitimately to be inferred from this fact — in the first place — that the average duration of human life was shorter in past time than it is nowadays; in the second, that the chances of error in accounts of alleged centenarians were infinitely greater — that if a man of eighty or eighty-five called himself one hundred or one hundred-and-ten, his story would be exceedingly apt to be believed.

